# Comprehensive analysis of RFC4 as a potential biomarker for regulating the immune microenvironment and predicting immune therapy response in lung adenocarcinoma

**DOI:** 10.3389/fimmu.2025.1578243

**Published:** 2025-06-19

**Authors:** Jianqing Zheng, Na Lin, Bifen Huang, Min Wu, Lihua Xiao, Bingwei Zeng

**Affiliations:** ^1^ Department of Radiation Oncology, The Second Affiliated Hospital of Fujian Medical University, Quanzhou, Fujian, China; ^2^ Department of Pathology, The Second Affiliated Hospital of Fujian Medical University, Quanzhou, Fujian, China; ^3^ Department of Obstetrics and Gynecology, People’s Hospital Affiliated of Quanzhou Medical College, Quanzhou, Fujian, China

**Keywords:** lung adenocarcinoma, replication factor C subunit 4 (RFC4), immune regulatory factors, tumor immune microenvironment, immune therapy response

## Abstract

**Background:**

Replication factor C subunit 4 (RFC4) is crucial for initiating DNA replication via DNA polymerase δ and ϵ and is overexpressed in various cancers. However, its relationship with the tumor immune microenvironment (TIME), and immunotherapy response in lung adenocarcinoma (LUAD) remains unclear. This study aimed to determine whether overexpressed RFC4 impacts survival in patients with LUAD and to explore potential mechanisms of RFC4 in regulating the TIME using integrated bioinformatics.

**Methods:**

LUAD gene expression data were downloaded from the Cancer Genome Atlas (TCGA) database and used for exploratory analysis. Differential expression of RFC4 was validated using gene expression data from the Gene Expression Omnibus (GEO). Clinical data with survival information from TCGA and GEO were use to explore and validate the prognostic value of RFC4. The relationship between RFC4 and TIME was studied by Cell-type identification by estimating relative subsets of RNA transcripts (CIBERSORT) and Estimation of Stromal and Immune cells in Malignant Tumor tissues using Expression data (ESTIMATE). Tumor Immune Dysfunction and Exclusion (TIDE) was used to predict the therapeutic response of RFC4 to immune checkpoint inhibitors. We validated the differential expression of RFC4 in LUAD and adjacent tissues using immunohistochemical staining in a real-world cohort from the Second Affiliated Hospital of Fujian Medical University.

**Results:**

RFC4 was significantly over-expressed in LUAD at both the RNA and protein levels. High RFC4 expression levels were associated with poor prognosis in LUAD, both in TCGA and GEO. High RFC4 levels were significantly associated with immunostimulators and immune cells infiltration in LUAD tissues. Correlation analysis revealed a significant relationship between the RFC4 and ESTIMATE scores. A high RFC4 expression level was associated with a lower TIDE score, indicating a stronger therapeutic response to immunotherapy. Functional prediction of RFC4 suggested that RFC4 mainly participated in DNA replication and repair, and reshaped the TIME.

**Conclusions:**

RFC4 proved to be a promising biomarker for tumorigenesis and could effectively predict immunotherapy response in LUAD. RCF4 altered tumor prognosis by reshaping the TIME, and targeted inhibition of RCF4 may be a promising new strategy for treating LUAD.

## 1 Introduction

Lung cancer (LC) is the leading cause of cancer-related deaths worldwide (1), with lung adenocarcinoma (LUAD) is the predominant histological subtype, accounting for approximately 40%-50% of all LC cases (2, 3). Most patients with LUAD are diagnosed at an advanced stage or have cancer metastasis, which results in a poor prognosis with a 5-year overall survival (OS) of <20% (4, 5). Recent significant progress has been made in immunotherapy with immune checkpoint inhibitors (ICIs) for LUAD, resulting in significantly reduced mortality rates (6). Owing to significant improvements in the clinical efficacy of immunotherapy for advanced LC, immunotherapy has become the preferred treatment mode for advanced LC (7). Several biomarkers have been widely used to predict immunotherapy response (IMTR) in clinical sets, including programmed death-ligand 1 (PD-L1) expression and tumor mutation burden (8). However, these biomarkers do not fully reflect the heterogeneity of the tumor microenvironment (TME) or the tumor immune microenvironment (TIME), and immunotherapy can only achieve remarkable clinical benefits in a few patients with cancer (9). Therefore, new biomarkers for predicting the prognosis and therapeutic efficacy of immunotherapy need to be identified.

The replication factor C subunit 4 (RFC4) gene encodes a highly conserved protein that is involved in many cellular processes related to DNA repair and DNA replication (10). RFC4 is necessary for DNA polymerase δ and DNA polymerase ϵ to extend primer DNA templates (11, 12). The RFC family (RFCs) plays a clamp loader role in DNA synthesis, loading proliferating cell nuclear antigen (PCNA) onto DNA through adenosine triphosphate (ATP)-dependent processes (13). During the S phase of DNA replication, RFC participates in cell cycle checkpoint control by activating polymerase assembly (14). After DNA damage occurs, they activate their mismatch and excision repair mechanisms by forming complexes with PCNA (15). Therefore, RFCs play a crucial role in DNA repair after DNA damage. RFC4 may play a crucial role in cancer cell survival, and because of its significant ability to regulate cell division and proliferation, it may be a promising target for cancer therapy (16, 17). Although emerging evidence has demonstrated that RFC4 plays an oncogene role in many human cancers, its expression patterns and functions in LUAD remain unclear. In this study, various bioinformatics tools were used to analyze RFC4 as a potential oncogene and therapeutic target in LUAD. The future development direction of this field is also discussed to provide evidence that is more in line with RFC4 as a promising biomarker in immunotherapy for LUAD.

## 2 Materials and methods

### 2.1 Data collection of LUAD samples

**Figure 1:**
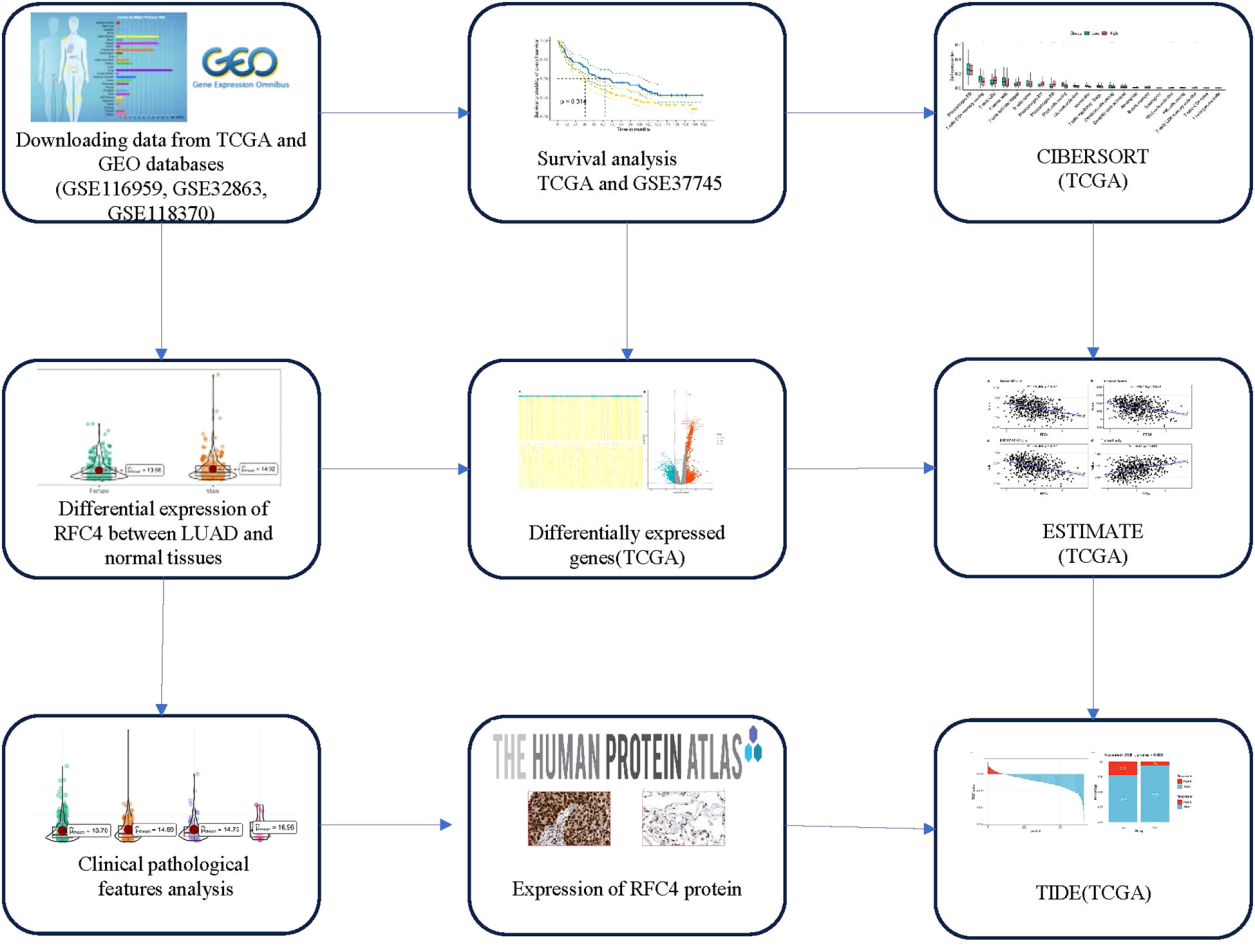
Flow chart of study design. TCGA, The cancer genome atlas; GEO, Gene expression omnibus; LUAD, lung adenocarcinoma; CIBERSORT, Cell-type identification by estimating relative subsets of RNA transcripts; ESTIMATE, Estimation of Stromal and Immune cells in Malignant Tumor tissues using Expression data; TIDE, Tumor Immune Dysfunction and Exclusion.

RNA sequencing (RNA-seq) data and clinical data were downloaded from the Cancer Genome Atlas (TCGA) (https://portal.gdc.cancer.gov/) and Gene Expression Omnibus (https://www.ncbi.nlm.nih.gov/geo/, GEO). A total of 586 samples were collected in the TCGA database, of which 527 were cancerous tissues and 59 were normal tissues. RNA-seq data was processed using standard bioinformatics procedures and used for subsequent analysis. The study flowchart is shown in **Figure 1**.

We validated the exploration results of TCGA with the GEO data. The inclusion criteria of the GEO data in our study were as follows: (1) datasets involving LUAD samples; (2) datasets with RNA-seq or gene microarrays from any type of sequencing platform; (3) datasets with normal tissues, which can be used to verify the differential expression of RFC4; and (4) datasets with clinical survival information, which can be used to verify the prognostic value of RFC4. The exclusion criteria for the GEO datasets were as follows: (1) datasets containing non-LUAD samples and (2) datasets without survival data and normal tissues. Finally, three external cohorts downloaded from GEO database were used to further validate the differential expression of RFC4: GSE116959 (18), GSE32863 (19), GSE118370 (20). One external cohort, GSE37745 (21), was used to further validate the prognostic efficacy of RFC4.

### 2.2 Differential expressions of RFC4 mRNA and protein between LUAD tissues and adjacent tissues or normal lung tissues

The Wilcox test was used to compare differential expression of RFC4 mRNA between LUAD tissues and adjacent tissues or normal lung tissues in TCGA and GEO datasets. Subsequently, we applied the “ggplot2” R package to show the results. The Human Protein Atlas (HPA, http://www.proteinatlas.org) was used to explore the protein expression levels of RFC4.

### 2.3 Survival analysis and clinical correlation analysis of RFC4

Clinicopathological features and survival data were extracted from the TCGA and GEO datasets (GSE37745). The relationship between RFC4 mRNA and different clinicopathological characteristics, such as survival status, cancer status, age, gender, race, and clinical stage, was explored using an independent sample Wilcox test or one-way analysis of variance. Using the best cutoff value of RFC4 mRNA in cancer tissues, patients with LUAD were divided into high expression (RFC4^High^) and low expression (RFC4^Low^) groups. The Kaplan-Meier method was used to plot the OS curves of the two groups, and log-rank test was used to compare difference. Next, survival results were further validated in patients with LUAD and then divided using the same method. Survival was analyzed using the “survival,” “survminer,” and “forestplot” packages.

### 2.4 Screening and functional analysis of RFC4 related differentially expressed genes in LUAD

Using the median value of RFC4 mRNA in TGCA, the patients were divided into high expression (RFC4^High^) and low expression (RFC4^Low^) groups. Subsequently, the “limma” package was used to identify DEGs in cancer tissues between the RFC4^High^ and the RFC4^Low^ groups. The top 50 DEGs closest to RFC4 were selected, and a heatmap was plotted using the “pheatmap” package. RFC4-related DEGs were selected to perform Gene Ontology (GO) and Kyoto Encyclopedia of Genes and Genomes (KEGG) analysis using the R package “clusterProfiler”. GO analysis of cell composition, biological processes, and molecular function was performed using the enrichGO function in the “clusterProfiler” R package. KEGG analysis was performed using the enrichKEGG function in the “clusterProfiler” R package. Pathways with *P* < 0.05 were considered significantly enriched.

### 2.5 Immune cell infiltration analysis and gene set variation analysis

The correlation between RFC4 expression and various infiltrating immune cells in the TIME was explored and analyzed by Spearman correlation analysis. Significance was set at *P*<0.05. Gene sets of immune-regulatory factors, including immunoinhibitors and immunostimulators, were screened from previously reported references (22–24). Correlation analyses between various immunoregulatory factors and RFC4 expression were displayed using lollipop plots. To simplify interpretation, we separately analyzed immunoinhibitors and immunostimulators using the “GSVA” package.

Cell-type identification by estimating relative subsets of RNA transcripts (CIBERSORT) was used to analyze the infiltration of immune cells between the RFC4^High^ and the RFC4^Low^ groups (25). CIBERSORT can obtain the infiltrating characteristics of 22 immune cell types with gene expression profiles and provide changes in characteristics of TIME in different cancer tissues.

### 2.6 Estimation of stromal and immune scores

The Estimation of Stromal and Immune cells in Malignant Tumor tissues using Expression data (ESTIMATE) was used to predict tumor purity and stromal/immune cell infiltration (26), which assess levels of stromal and immune cell infiltration using expression profiles by the “estimate” R package. Stromal, immune, ESTIMATE, and tumor purity scores were calculated using RNA sequencing data from TCGA cohort. A Wilcoxon test was then performed to compare scores between the two groups.

### 2.7 Immunotherapy response prediction

Tumor Immune Dysfunction and Exclusion (TIDE) (http://tide.dfci.harvard.edu/) was used to predict response to immune checkpoint blockade therapy. The Wilcoxon test was performed to compare TIDE scores between the two groups.

### 2.8 RFC4 protein expression in LUAD

The RFC4 protein expression in LUAD tissues was evaluated by immunohistochemical staining in both cancer tissues and normal tissues, and the data was retrieved and downloaded from the Human Protein Atlas database (HPA, http://www.proteinatlas.org/).

To verify the differential expression of RFC4 protein, we recruited 31 patients with LUAD who underwent surgical treatment at the Second Affiliated Hospital of Fujian Medical University between January 2021 and May 2024. Postoperative cancer tissues were donated by the patient or their family members, and written informed consent was obtained. This study has been approved by the Ethics Committee of the Second Affiliated Hospital of Fujian Medical University (2025-001). RFC4 rabbit polyclonal antibody was purchased from Wuhan Sanying Company. Immunohistochemical (IHC) staining was conducted by Na Lin, and the results were interpreted by two members of the research team (Bingwei Zeng and Jianqing Zheng).

### 2.9 Statistical analysis

Statistical analysis of the association between RFC4 and clinicopathological parameters was performed using independent sample t-test, Wilcoxon test, chi-square test, or Fisher’s exact test. For survival variables, Kaplan-Meier curves were plotted as well as log-rank tests. The prognostic value of RFC4 was analyzed using the Cox proportional risk model via “survminer” and “survival” R package. Significance was set at P<0.05. The above analyses were performed using the R software (version 4.3.1).

## 3 Results

### 3.1 Expression of RFC4 mRNA in LUAD tissues

**Figure 2:**
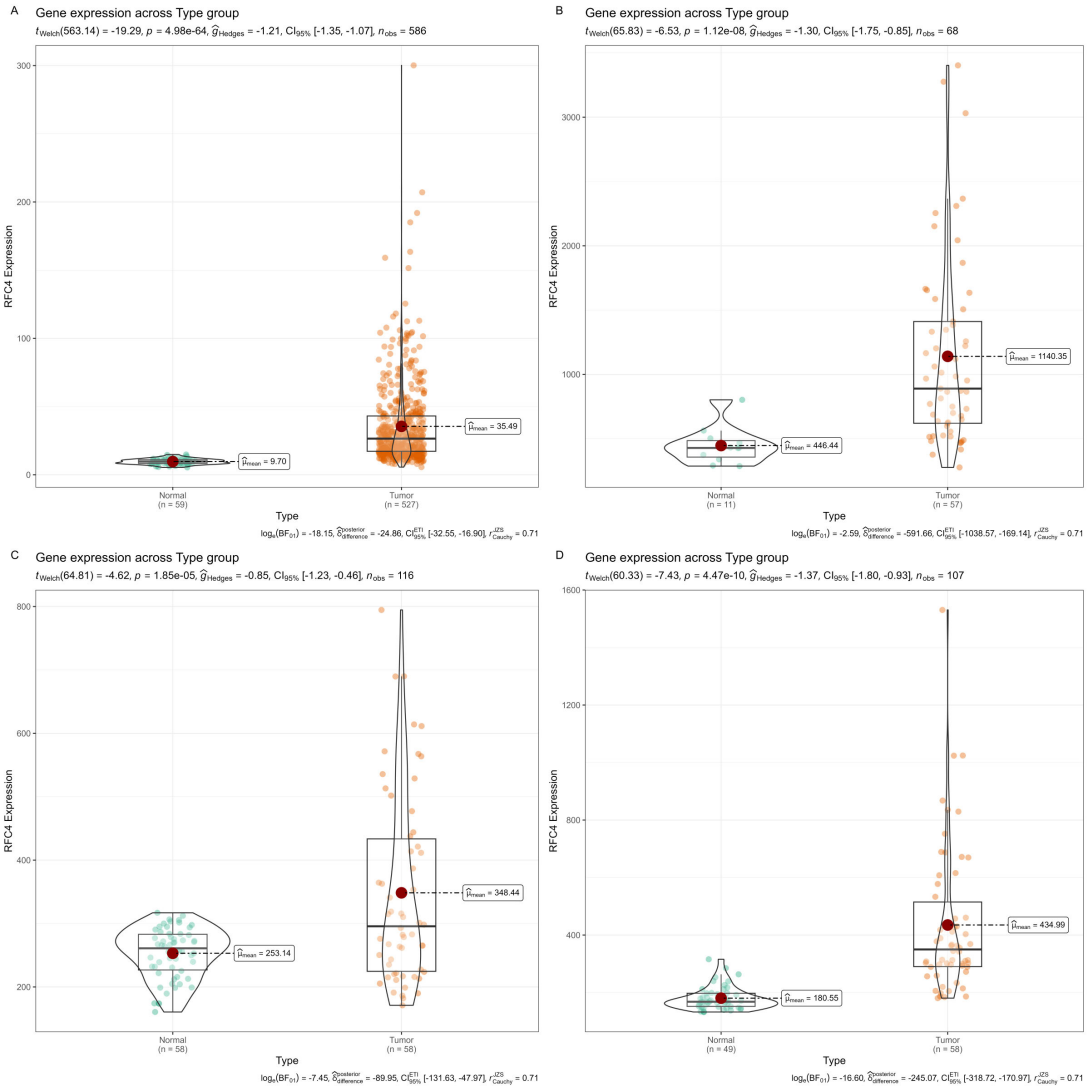
Expression of RFC4 mRNA in LUAD tissues from TCGA and GEO. **(A)** Expression of RFC4 mRNA between cancer tissues and normal tissue in TCGA cohort. **(B)** Expression of RFC4 mRNA between cancer tissues and normal tissue in GSE116959 cohort. **(C)** Expression of RFC4 mRNA between cancer tissues and normal tissue in GSE32863 cohort. **(D)** Expression of RFC4 mRNA between cancer tissues and normal tissue in GSE118370 cohort.

The differential expression of RFC4 mRNA between LUAD cancer and normal tissue samples is shown in **Figures 2A-D**. RFC4 expression level was significantly higher in LUAD tissues than normal tissues in TCGA samples(*P*<0.001). The differential expression of RFC4 in LUAD was further validated using the GEO dataset. Consistent results in the exploration and validation sets indicated that RFC4 mRNA expression was significantly elevated in LUAD samples, suggesting the involvement of RFC4 in LUAD tumorigenesis.

### 3.2 Relationship between RFC4 and clinical characteristics of patients

**Figure 3:**
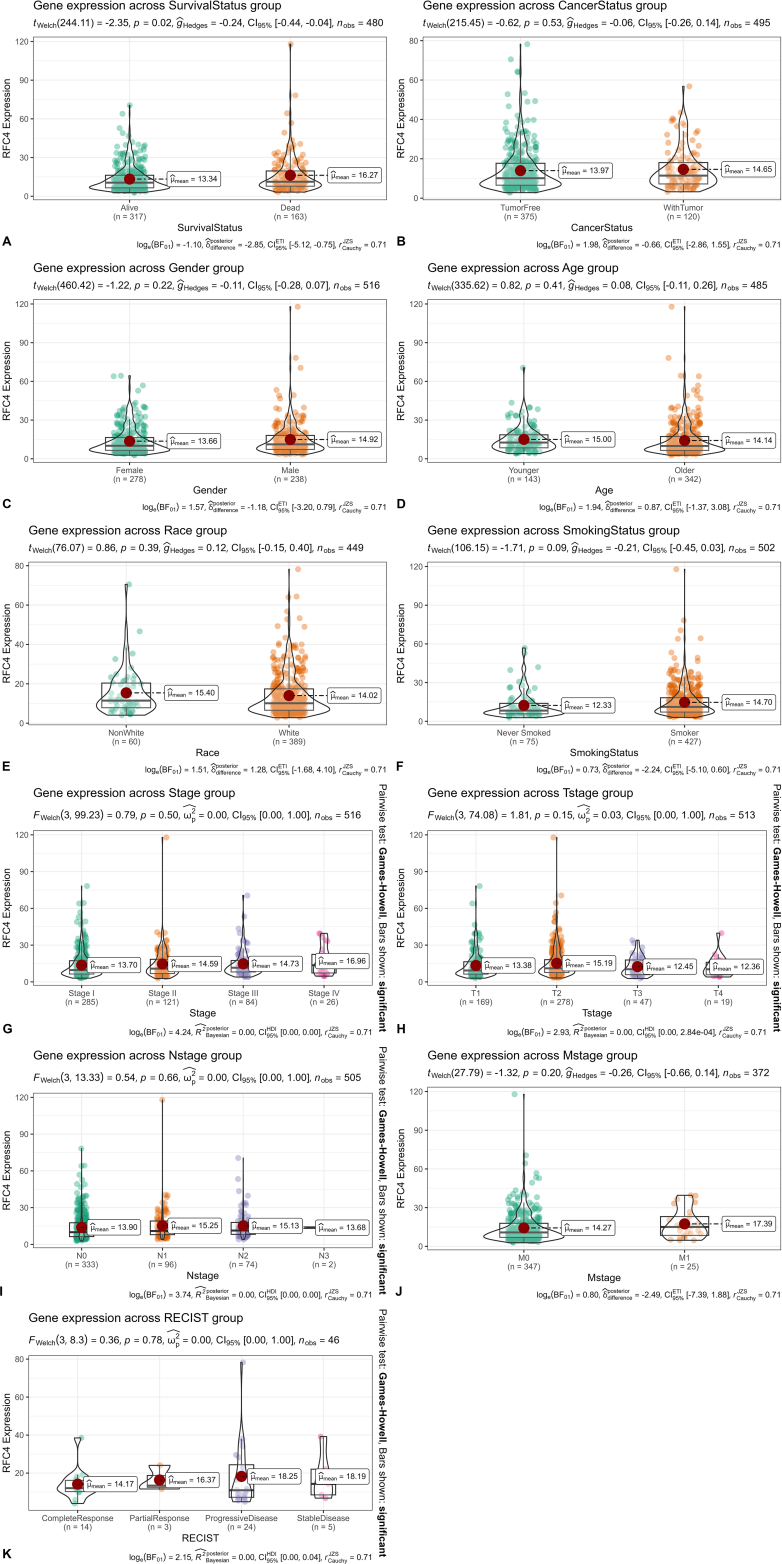
Relationship between RFC4 mRNA and clinical characteristics of patients. **(A)** Expression of RFC4 mRNA with different survival status (Alive: Patients who still lived in the TCGA samples. Dead: Patients who have died in the TCGA samples.). **(B)** Expression of RFC4 mRNA with different cancer status (WithTumor: Patients who still lived or died with tumor. TumorFree: Patients who still lived or died without tumor). **(C)** Expression of RFC4 mRNA with different gender. **(D)** Expression of RFC4 mRNA with different survival status. **(A)** Expression of RFC4 mRNA with different age (Younger: <60 years old, Older:≥60 years old). **(E)** Expression of RFC4 mRNA with different race (white and non-white). **(F)** Expression of RFC4 mRNA with different smoking status (smoker and never smoked). **(G)** Expression of RFC4 mRNA with different clinical stage. **(H)** Expression of RFC4 mRNA with different T stage. **(I)** Expression of RFC4 mRNA with different N stage. **(J)** Expression of RFC4 mRNA with different M stage. **(K)** Expression of RFC4 mRNA with different RECIST status.

We divided the TCGA samples into different groups based on the following clinical characteristics, survival status (alive: patients who still lived in the TCGA samples. dead: patients who have died in the TCGA samples.), cancer status (WithTumor: patients who still lived or died with tumor. TumorFree: patients who still lived or died without tumor), gender (males and females), age (younger: <60 years old, older:≥60 years old), race (white and non-white), smoking status (smoker and never smoked), clinical stage (stage I, stage II, stage III, and stage IV). According to the Response Evaluation Criteria in Solid Tumors (RECIST), the samples were divided into complete response, partial response, progressive disease and stable disease groups. The results of the RFC4 expression in LUAD samples from different groups are shown in **Figures 3A–K**. Only survival status demonstrated a significant relationship with RFC4 expression.

### 3.3 Relationship between RFC4 and prognosis of patients with LUAD

**Figure 4:**
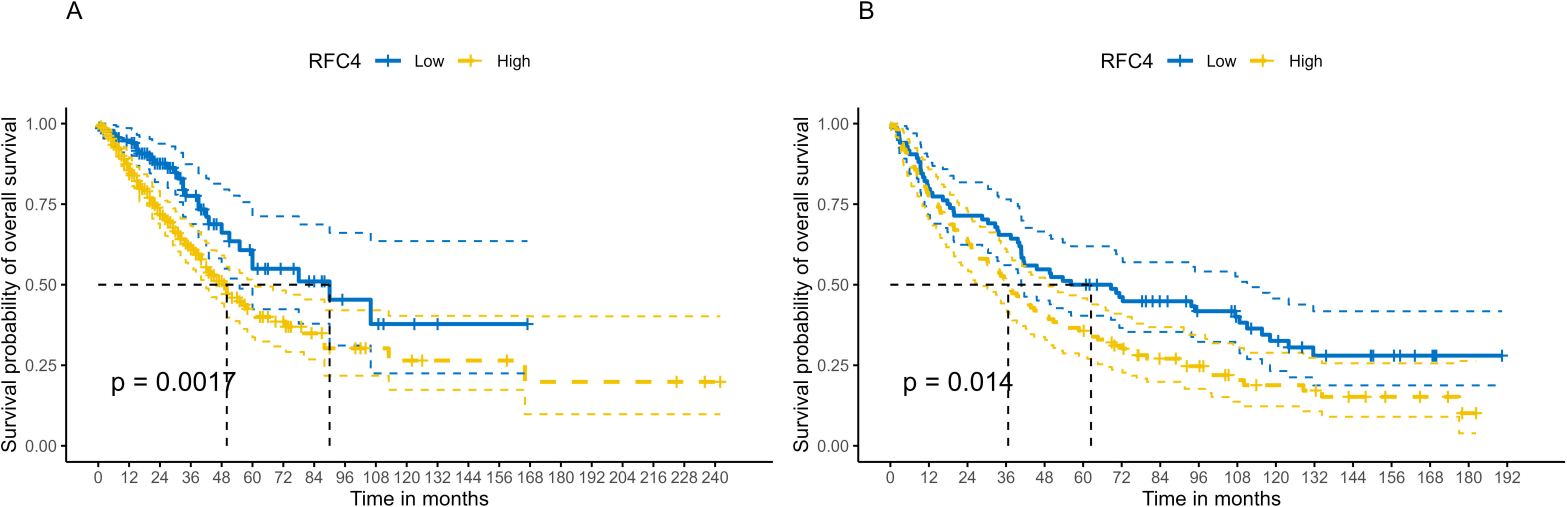
Kaplan–Meier survival analysis of RFC4 expression on survival in LUAD. **(A)** Overall survival from TCGA. **(B)** Validation result of survival from GSE37745 dataset.

Using best cutoff value of 7.16, patients with LUAD in the TCGA database were divided into the RFC4^High^ group (n=344) and RFC4^Low^ group (n=136). Kaplan-Meier survival analysis showed that patients with high RFC4 expression levels had significantly worse OS than those with low RFC4 expression levels (hazard ratio [HR] =1.83; 1.25-2.68, *P*=0.002) (**Figure 4A**). The 5-year survival rate was 54.95% (42.39%-71.23%) in the RFC4^Low^ group and 41.25% (33.68%-50.51%) in the RFC4 ^High^ group, respectively. Using best cutoff value of 9.31, patients with LUAD in the GSE37745 dataset were divided into the RFC4^High^ group (n=112) and RFC4^Low^ group (n=84). Kaplan-Meier survival analysis showed that patients with high RFC4 expression had worse overall survival than those with low RFC4 expression levels had significantly worse OS than those with low RFC4 expression levels (HR = 1.52; 1.09–2.13, *P*=0.015) (**Figure 4B**). 5-year survival rate was 50.00% (40.37%-61.92%) in the RFC4^Low^ group, and 35.71% (27.86%-45.79%) in RFC4 ^High^ group, respectively.

To verify whether RFC4 has an independent prognostic value, multivariate analysis was conducted. Univariate analysis showed that the RFC4 expression, clinical stage, cancer status and residual tumor were potential factors for the OS of patients with LUAD (*P*<0.05), as shown in **Table 1**. A multivariate COX regression analysis based on the abovementioned four positive variables was performed, and the results were presented in **Table 2**. In the stepwise regression multivariate model, RFC4.AutoCut significantly affected the OS (HR=1.52, 95%CI: 1.09-2.12, *P*=0.007), thus suggesting the independent prognostic value of RFC4 in LUAD.

**Table 1:** Univariate analysis of the prognostic ability of RFC4 in patients with LUAD.

Characteristics	Levels	Beta	SE	HR (95% CI for HR)	Statistics (Z value)	P
RFC4		0.01	0.01	1.01 (1.00, 1.02)	2.496	0.013
RFC4.Median	Low					
	High	0.27	0.15	1.30 (0.97, 1.76)	1.731	0.083
RFC4.AutoCut	Low					
	High	0.56	0.17	1.75 (1.26, 2.43)	3.360	<0.001
CancerStatus	TumorFree					
	WithTumor	1.45	0.15	4.28 (3.16, 5.80)	9.412	<0.001
Gender	Female					
	Male	0.06	0.15	1.06 (0.79, 1.44)	0.403	0.687
Age_group	Younger					
	Older	0.11	0.17	1.12 (0.80, 1.56)	0.673	0.501
SmokingStatus	Never Smoked					
	Smoker	-0.03	0.21	0.97 (0.64, 1.47)	-0.140	0.889
TumorSite	L-Lower					
	L-Upper	0.08	0.25	1.08 (0.67, 1.75)	0.316	0.752
	R-Lower	0.24	0.25	1.27 (0.77, 2.08)	0.949	0.343
	R-Middle	0.24	0.46	1.27 (0.52, 3.11)	0.531	0.595
	R-Upper	-0.12	0.24	0.89 (0.56, 1.42)	-0.500	0.617
ResidualTumor	R0					
	R1/R2	1.43	0.26	4.19 (2.54, 6.93)	5.593	<0.001
	Rx	0.27	0.37	1.31 (0.64, 2.68)	0.736	0.462
Stage	Stage I					
	Stage II	0.96	0.19	2.62 (1.79, 3.83)	4.966	<0.001
	Stage III	1.38	0.20	3.98 (2.71, 5.85)	7.024	<0.001
	Stage IV	1.50	0.28	4.47 (2.56, 7.80)	5.271	<0.001

**Table 2:** Multivariate analysis of prognostic ability of RFC4 in patients with LUAD.

Characteristics	Levels	Beta	SE	HR (95% CI for HR)	Statistics (Z value)	P
RFC4.AutoCut	Low					
	High	0.42	0.17	1.52 (1.09,2.12)	2.444	0.015
CancerStatus	TumorFree					
	WithTumor	1.16	0.17	3.18 (2.28,4.42)	6.854	<0.001
ResidualTumor	R0					
	R1/R2	0.73	0.28	2.07 (1.21,3.56)	2.650	0.008
	Rx	-0.09	0.37	0.92 (0.45,1.89)	0.238	0.812
Stage	Stage I					
	Stage II	0.68	0.20	1.96 (1.33,2.90)	3.399	0.001
	Stage III	1.11	0.20	3.04 (2.05,4.52)	5.505	<0.001
	Stage IV	0.66	0.30	1.93 (1.06,3.50)	2.166	0.030

### 3.4 Analysis of DEGs and functional enrichment related to RFC4

**Figure 5:**
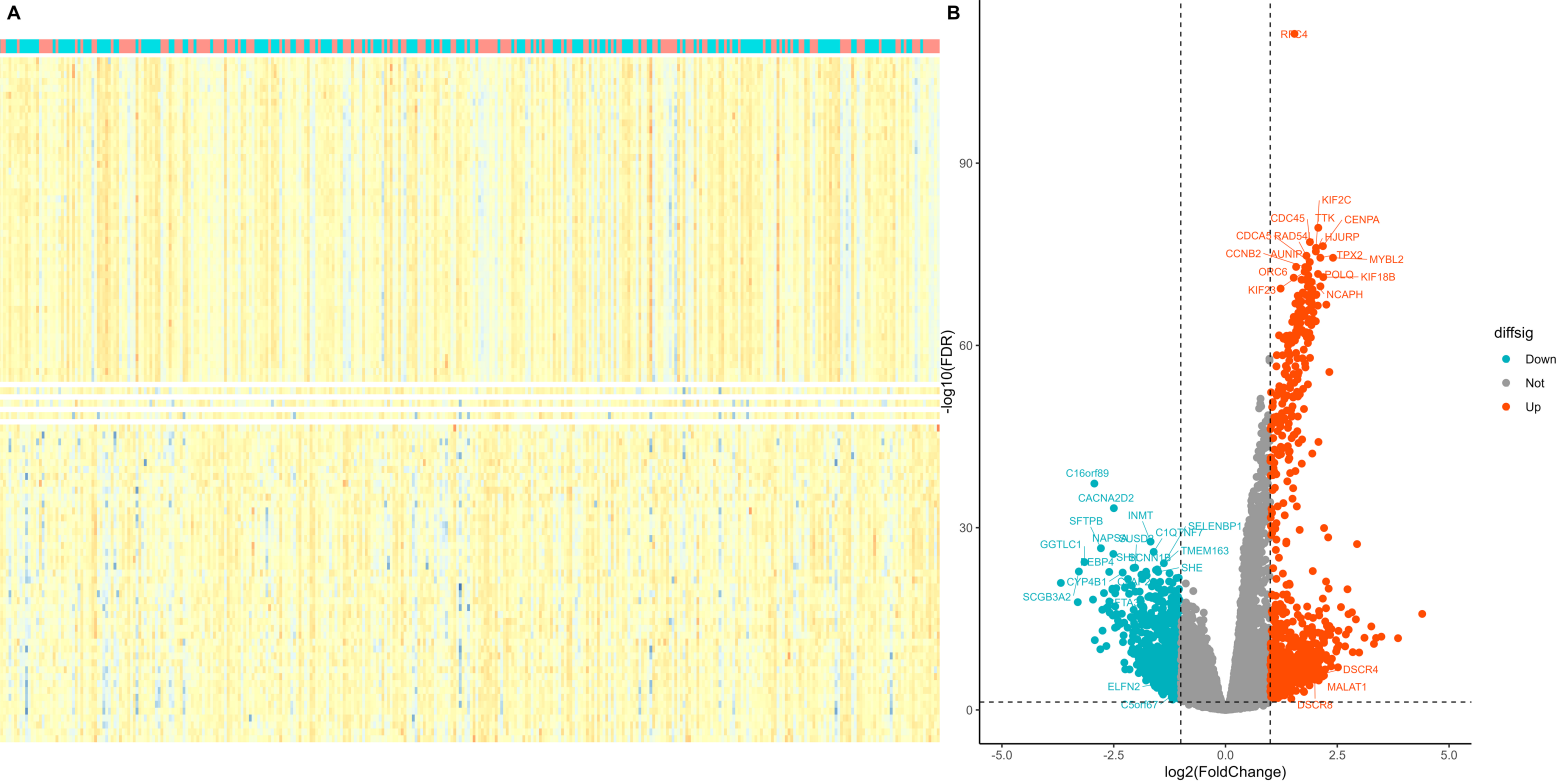
Screening of differentially expressed genes in different RFC4 status in LUAD cohort. **(A)** Heatmap. The figure shows 50 genes with the most significant upregulation, 50 genes with the most significant downregulation. **(B)** volcano plot. Differentially expressed genes were selected to labelled.

Using the median expression value of RFC4, patients with LUAD in the TCGA database were divided, and a differential expression analysis was conducted. Using the absolute value of log fold change≧1 and *P <*0.05 as screening criteria, a total of 1346 DEGs were identified, of which 746 genes were highly expressed and 600 genes were lowly expressed. Detailed information of DEGs were listed in **Supplementary Table S1**. Heatmaps and volcano maps are provided in **Figures 5A, B**, respectively.

**Figure 6:**
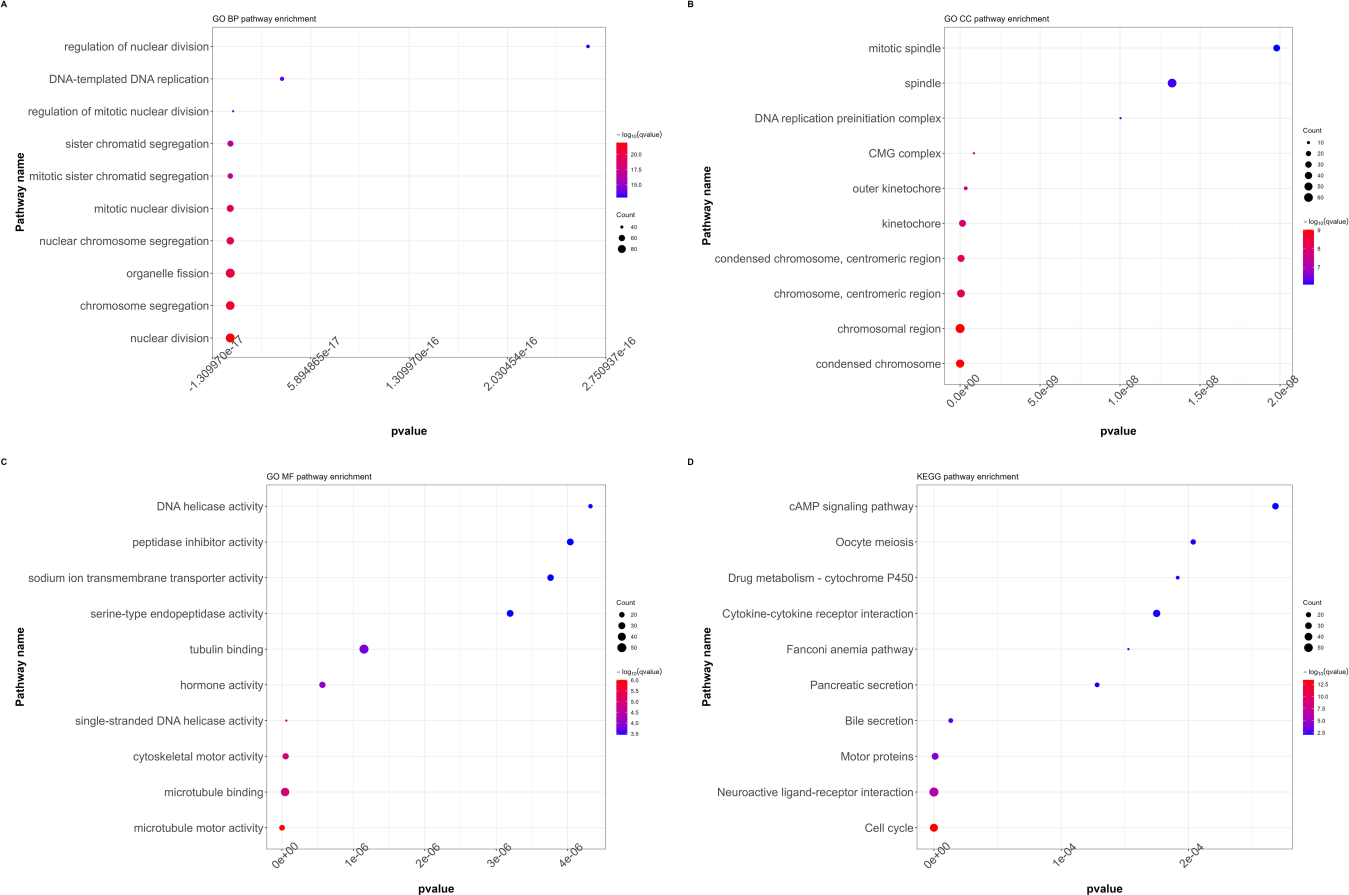
Functional annotation and pathway enrichment analysis of differentially expressed genes in LUAD cohort. **(A)** GO functional annotation (BP, biological processes). **(B)** GO functional annotation (CC, cellular composition). **(C)** GO functional annotation (MF, Molecular functions). **(D)** KEGG pathway enrichment. (GO, Gene Ontology; KEGG, Kyoto Encyclopedia of Genes and Genomes).

All DEGs that showed significant differences between the RFC4^High^ and RFC4^Low^ groups were screened and selected for functional enrichment analyses. The biological processes were mainly enriched in nuclear division, chromosome segregation, organelle fission, nuclear chromosome segregation, mitotic nuclear division, mitotic sister chromatid segregation, sister chromatid segregation, regulation of mitotic nuclear division, DNA-templated DNA replication, and regulation of nuclear division. The cellular composition was mainly enriched in condensed chromosomes, chromosomal regions, chromosomes, centromeric regions, condensed chromosomes, centromeric regions, kinetochores, outer kinetochores, CMG complexes, DNA replication pre-initiation complexes, spindles, and mitotic spindles. The molecular functions were mainly enriched for microtubule motor activity, microtubule binding, cytoskeletal motor activity, single-stranded DNA helicase activity, hormone activity, tubulin binding, serine-type endopeptidase activity, sodium-ion transmembrane transporter activity, peptidase inhibitor activity, and DNA helicase activity. The KEGG pathways were mainly enriched in the cell cycle, neuroactive ligand–receptor interaction, motor proteins, bile secretion, pancreatic secretion, Fanconi anemia pathway, cytokine–cytokine receptor interaction, drug metabolism-cytochrome P450, oocyte meiosis, and the cAMP signaling pathway. The results of the functional enrichment analysis are shown in **Figures 6A–D**. Detailed information on the functional enrichment analysis is provided in **Supplementary Table S2**.

### 3.5 Correlation analysis between RFC4 and immunostimulators and immunoinhibitors

**Figure 7:**
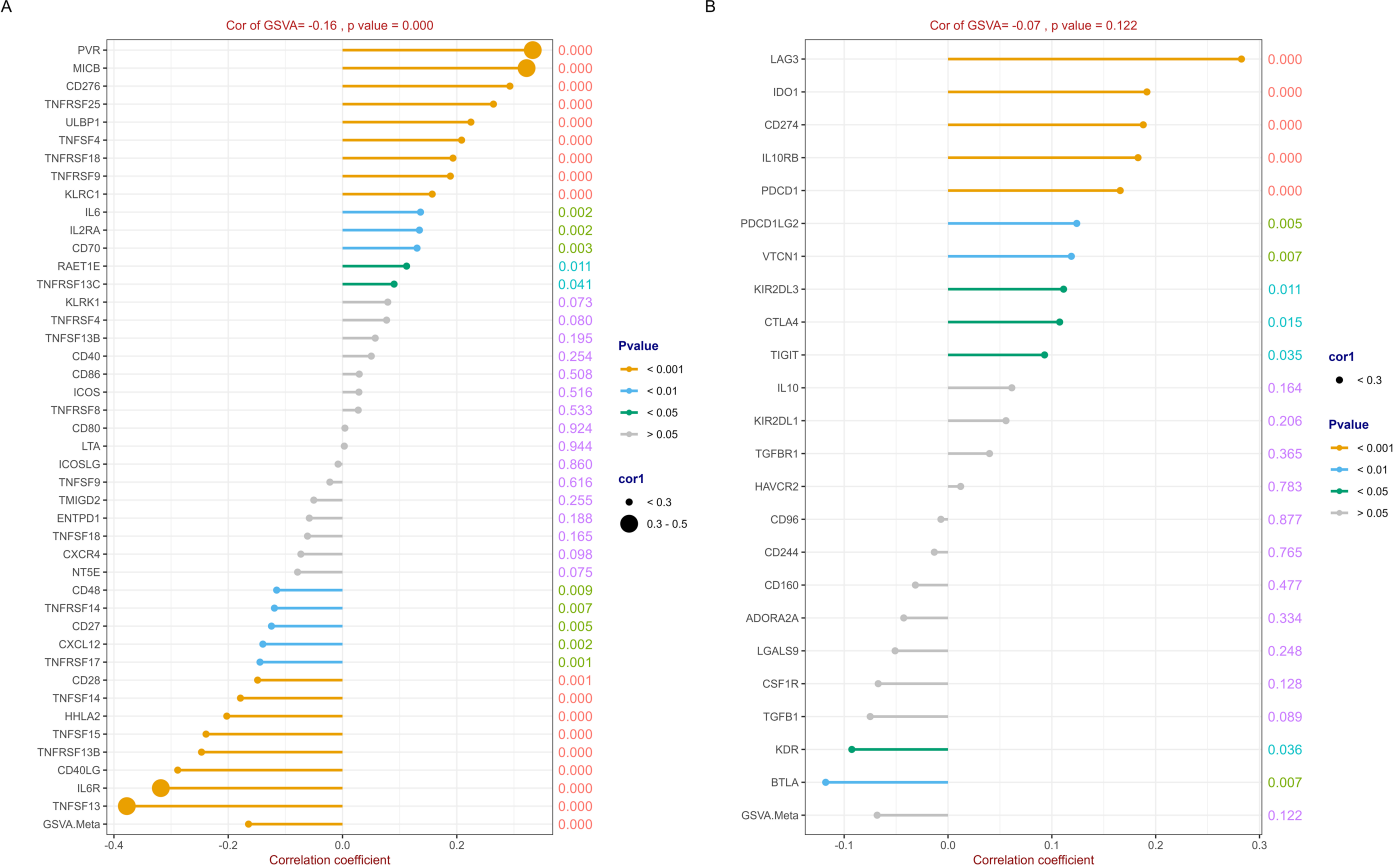
Correlation analysis between RFC4 and immunostimulators and immunoinhibitors. **(A)** Correlation analysis of 43 immunostimulators with RFC4. **(B)** Correlation analysis of 23 immunoinhibitors with RFC4. (Correlation analysis was conducted using Pearson’s method. The numbers on the right side of each line represent the statistical significance of the correlation coefficient, that is, the P value. cor1: the abbreviation of the correlation coefficient).

The detail results of the correlation analysis between RFC4 and the immunostimulators were listed in **Supplementary Table S3** and shown in **Figure 7A**. In our study, 43 immunostimulators were selected for correlation analysis. RFC4 expression was positively correlated with 14 immunostimulatory factors and negatively correlated with 13 immunostimulatory factors. We used GSVA to evaluate the correlation between RFC4 and immunostimulators and provided a correlation coefficient indicator called GSVA.Meta, which reflects the GSVA results. The GSVA results showed that RFC4 was negatively correlated with GSVA.Meta (rho=−0.164, *P*<0.001). Based on these results, we inferred that RFC4 mainly altered the TIME by suppressing the expression of immune-stimulatory factors.

Similarly, 23 immunoinhibitors were selected for the correlation analysis. The detail results were listed in **Supplementary Table S4** and shown in **Figure 7B**. RFC4 expression was positively correlated with the expression of 10 immunosuppressive factors and negatively correlated with the expression of two immunosuppressive factors. The GSVA results showed that RFC4 was negatively correlated with GSVA.Meta, with no statistical significance (rho=−0.068, *P*=0.122).

### 3.6 Relationship between RFC4 and immune cell infiltration

**Figure 8:**
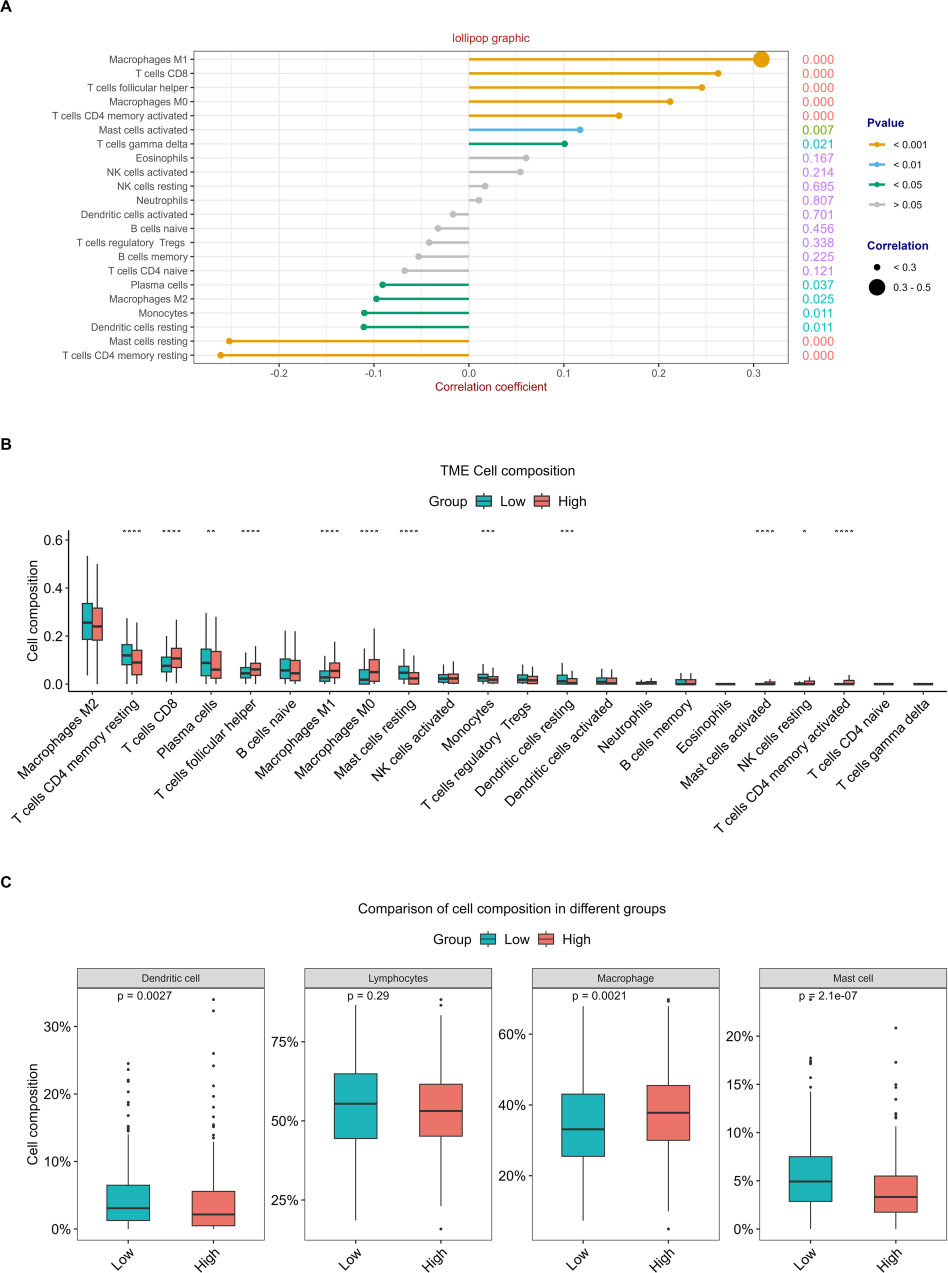
Analysis of tumor-infiltrating immune cells changes in different RFC4 status in LUAD cohort via CIBERSORT. **(A)** Correlation analysis of 22 immune cells with RFC4. **(B)** Differential analysis of immune cell infiltration between RFC4^High^ group and RFC4^Low^ group. **(C)** Four categories tumor-infiltrating immune cells between RFC4^High^ group and RFC4^Low^ group. (Correlation analysis was conducted using Pearson’s method. The numbers on the right side of each line represent the statistical significance of the correlation coefficient, that is, the P value. cor1: the abbreviation of the correlation coefficient). "*":<0.05, "**":<0.01, "***":<0.001 and "****":<0.0001.

To evaluate the discriminative potential of RFC4 for TIME and its applicability in immunotherapy in patients with LUAD, a correlation analysis between 22 immune cells and RFC4 was conducted using CIBERSORT, and results were shown in **Figure 8A**; **Supplementary Table S5**. Among them, T cells CD4 memory resting, mast cells resting, dendritic cells resting, monocytes, macrophages M2, and plasma cells were negatively correlated with RFC4 gene with significance, while T cells gamma delta, mast cells activated, T cells CD4 memory activated, macrophages M0, T cells follicular helper, T cells CD8, macrophages M1 were positively correlated with RFC4 gene with significance. The infiltration of 22 types of immune cells between RFC4^High^ group and RFC4^Low^ group was shown in **Figure 8B**. With median expression value of RFC4, our results showed that NK cells resting, macrophages M0, macrophages M1, mast cells activated, T cells CD4 memory activated, T cells CD8 and T cells follicular helper had more infiltration in the RFC4^High^ group, while plasma cells, dendritic cells resting, mast cells resting, monocytes and T cells CD4 memory resting had more infiltration in the RFC4^Low^ group. Furthermore, we regrouped immune cells into four categories, and dendritic cells, macrophages and mast cells showed the most significant differences between the RFC4^High^ group and RFC4^Low^ group, as shown in **Figure 8C**. Immune cell infiltration in different RFC4 groups and LUAD samples is shown in **Supplementary Figures S1A**, **S1B**.

**Figure 9:**
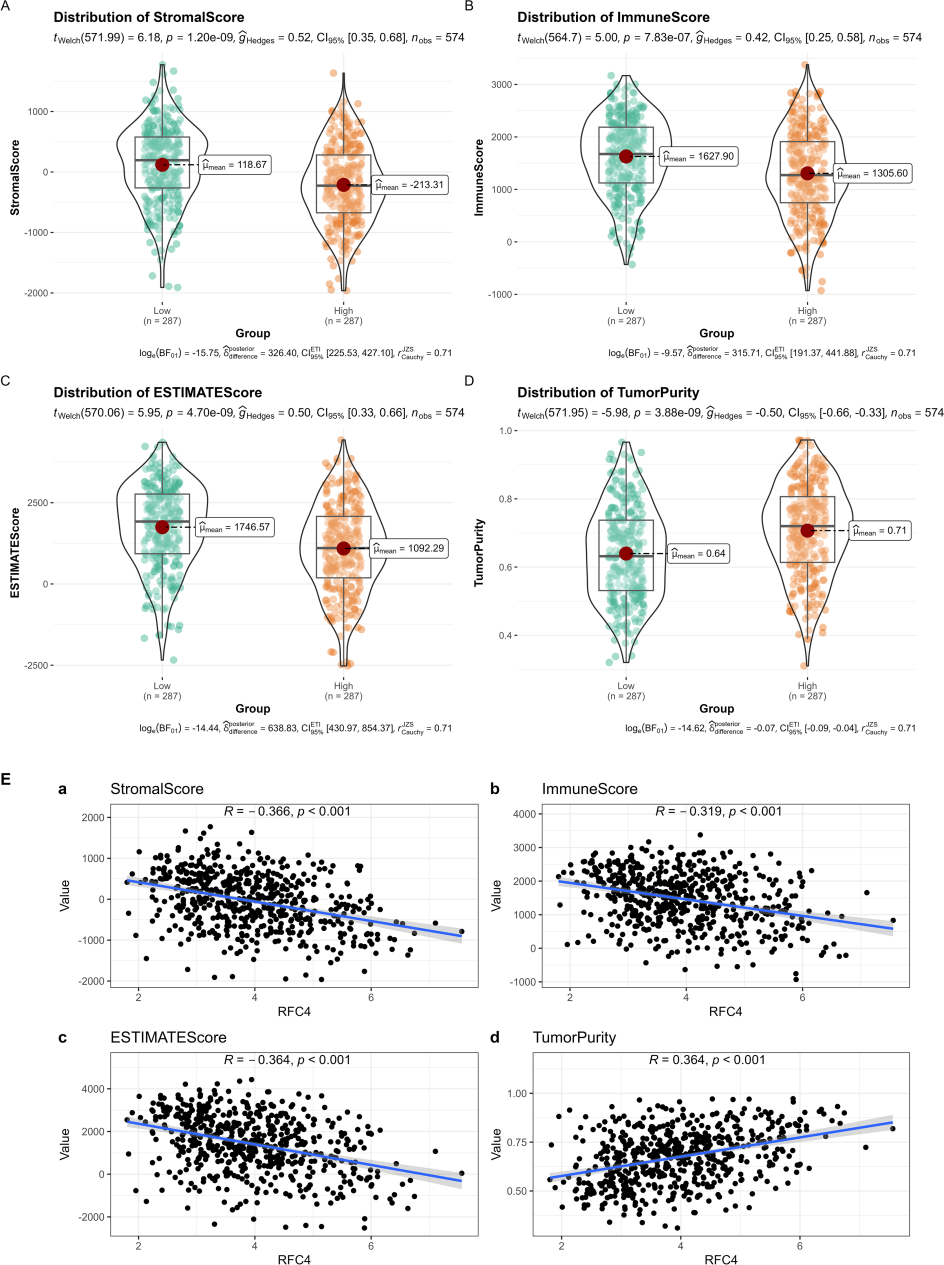
Analysis of tumor-infiltrating immune cells changes between RFC4^High^ group and RFC4^Low^ group in LUAD cohort via ESTIMATE. **(A)** Stromal scores. **(B)** immune scores. **(C)** ESTIMATE scores. **(D)** Tumor purity. **(E)** Correlation analysis between RFC4 and stromal scores **(a)**, immune scores **(b)**, ESTIMATE scores **(c)** and tumor purity **(d)**. Independent sample t-tests were used for the analysis of differences between groups in **(A–D)**. Correlation analysis was conducted using Pearson’s method in **(E)**.

The ESTIMATE analysis revealed that cancer tissues in the RFC4^High^ group had lower stromal scores, lower immune scores, lower ESTIMATE scores, and higher tumor purity than those in the RFC4^Low^ group, as shown in **Figures 9A–D**. Correlation analyses between RFC4 expression levels and stromal, immune, ESTIMATE, and tumor purity from ESTIMATE are shown in **Figure 9E**. Among them, stromal, immune and ESTIMATE scores were negatively correlated with RFC4 expression, whereas tumor purity was positively correlated with RFC4 expression.

### 3.7 Potential function prediction of RFC4

**Figure 10:**
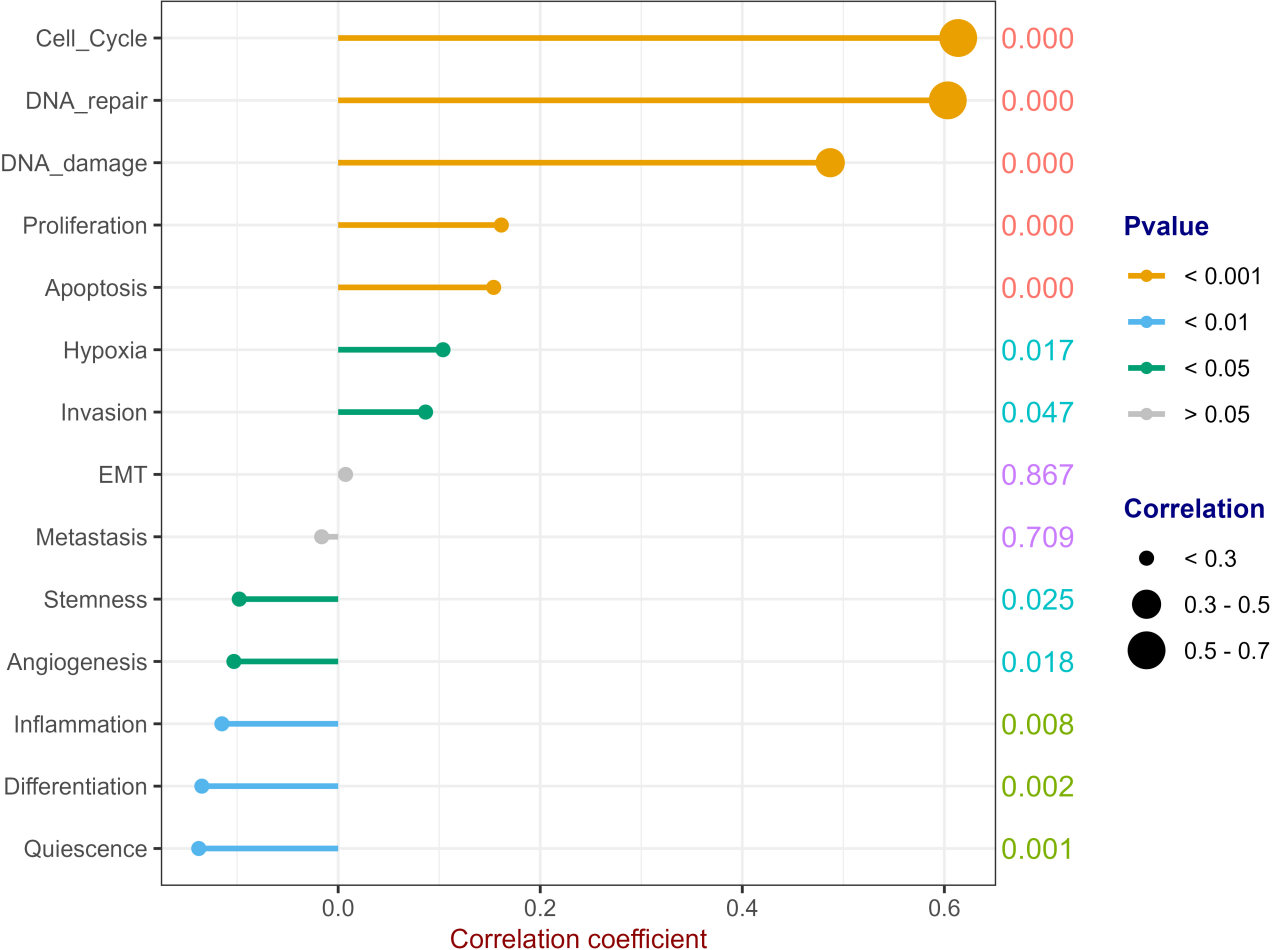
Potential Function Prediction of RFC4. (Correlation analysis was conducted using Pearson’s method. The numbers on the right side of each line represent the statistical significance of the correlation coefficient, that is, the P value. cor1: the abbreviation of the correlation coefficient).

We downloaded 14 gene sets with common cancer-related functions from CancerSEA (http://biocc.hrbmu.edu.cn/CancerSEA/goDownload) and used GSVA to predict the RFC4 function (27). Except epithelial-mesenchymal transition (EMT) and metastasis, RFC4 was widely involved in other biological processes, including angiogenesis, apoptosis, cell cycle, differentiation, DNA damage, DNA repair, hypoxia, inflammation, invasion, proliferation, quiescence and stemness, as shown in **Figure 10**. Among them, RFC4 has the strongest positive relationship with cell cycle, DNA repair and DNA damage, indicating that RFC4 was mainly involved in these biological processes.

### 3.8 Prediction of immunotherapy efficacy of RFC4 in LUAD

**Figure 11:**
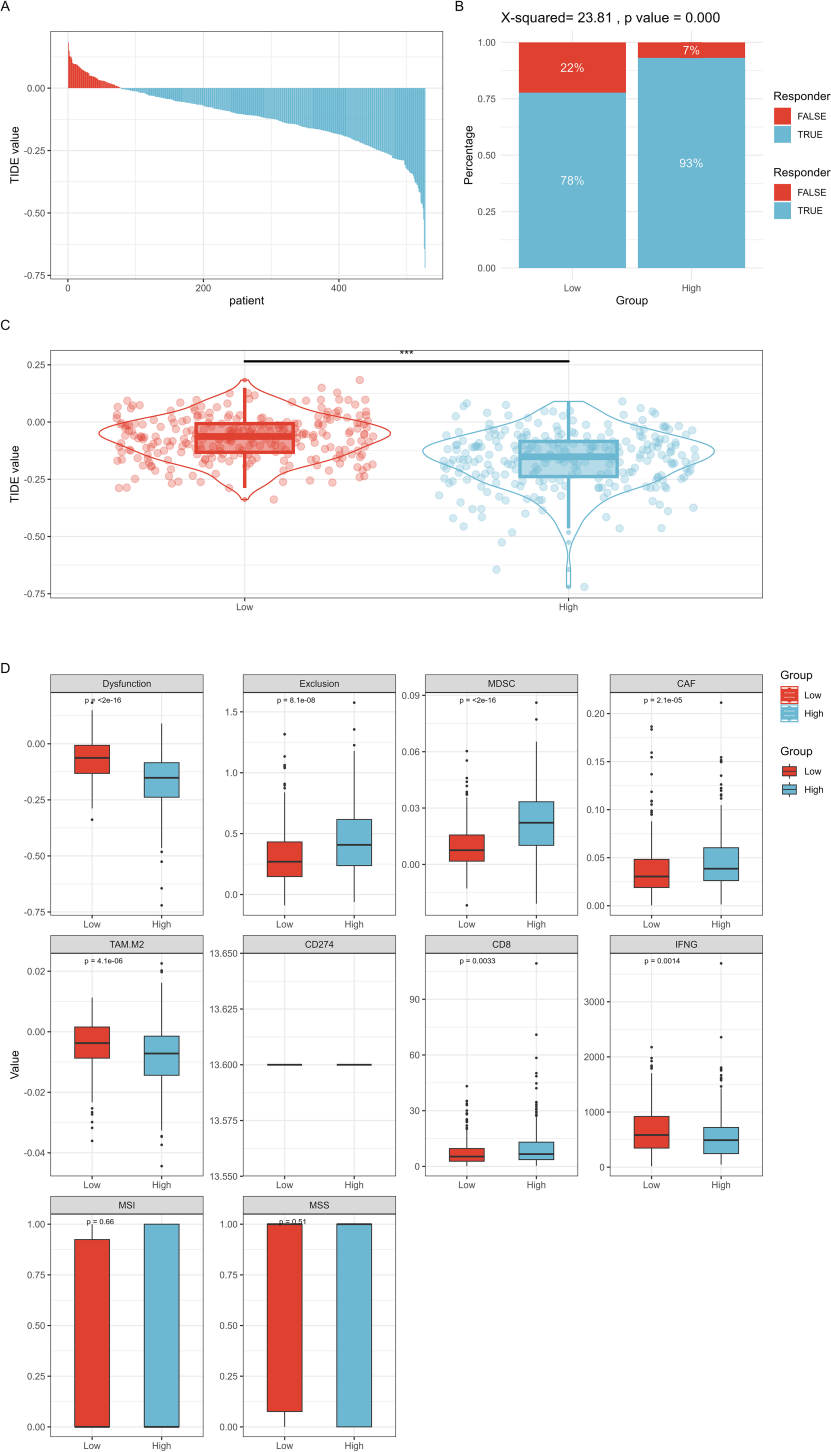
TIDE method predicted the efficacy of RFC4 in immunotherapy. **(A)** TIDE value of all TCGA samples. **(B)** Comparison of immune therapy response rates at different RFC4 expression levels. **(C)** Comparison of TIDE scores at different RFC4 expression levels. **(D)** Other immune therapy response prediction scores. "***":<0.001.

TIDE predicted the efficacy of RFC4 in immunotherapy, as shown in **Figures 11A–D**. The RFC4^High^ group showed a higher response rate to immune therapy than the RFC4^Low^ group. Lower TIDE scores were observed in the RFC4^High^ group, indicating higher immune sensitivity and increased patient benefit from ICIs treatment. Moreover, high RFC4 expression levels were correlated with lower dysfunction and higher exclusion. Furthermore, high RFC4 expression levels were correlated with higher MDSC, higher CAF, higher CD8 scores and lower TAM.M2 and lower IFNG scores. Thus, high RFC4 expression levels were correlated with better immunotherapy sensitivity in patients with LUAD.

### 3.9 RFC4 protein expression in LUAD

**Figure 12:**
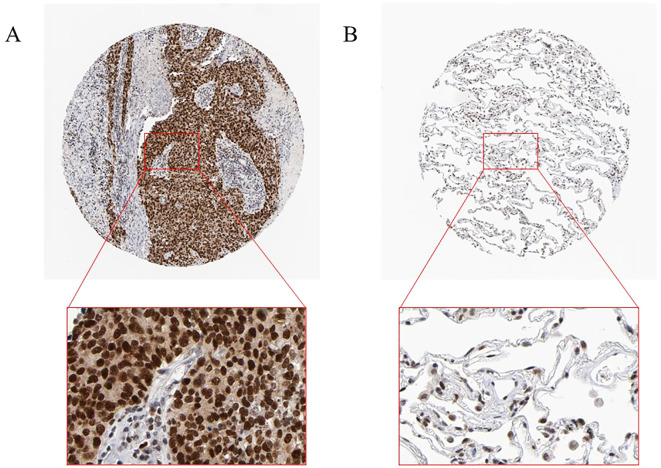
Interpretation of RFC4 in immunohistochemical staining. **(A)** High expression of RFC4 in LUAD cancer tissue. **(B)** Low expression of RFC4 in normal tissue.

To verify RFC4 protein expression in LUAD tissues, we analyzed IHC images from the HPA database. RFC4 protein exhibited moderate-to-strong expression in 83.33% (25/30) cases of LUAD tissues and 22.22% (2/9) case of normal tissues, and the difference was statistically significant (χ^2^ = 12.56, *P*=0.002). The IHC schematic of RFC4 protein in LUAD and normal lung tissues from the HPA database is shown in **Figures 12A, B**.

**Figure 13:**
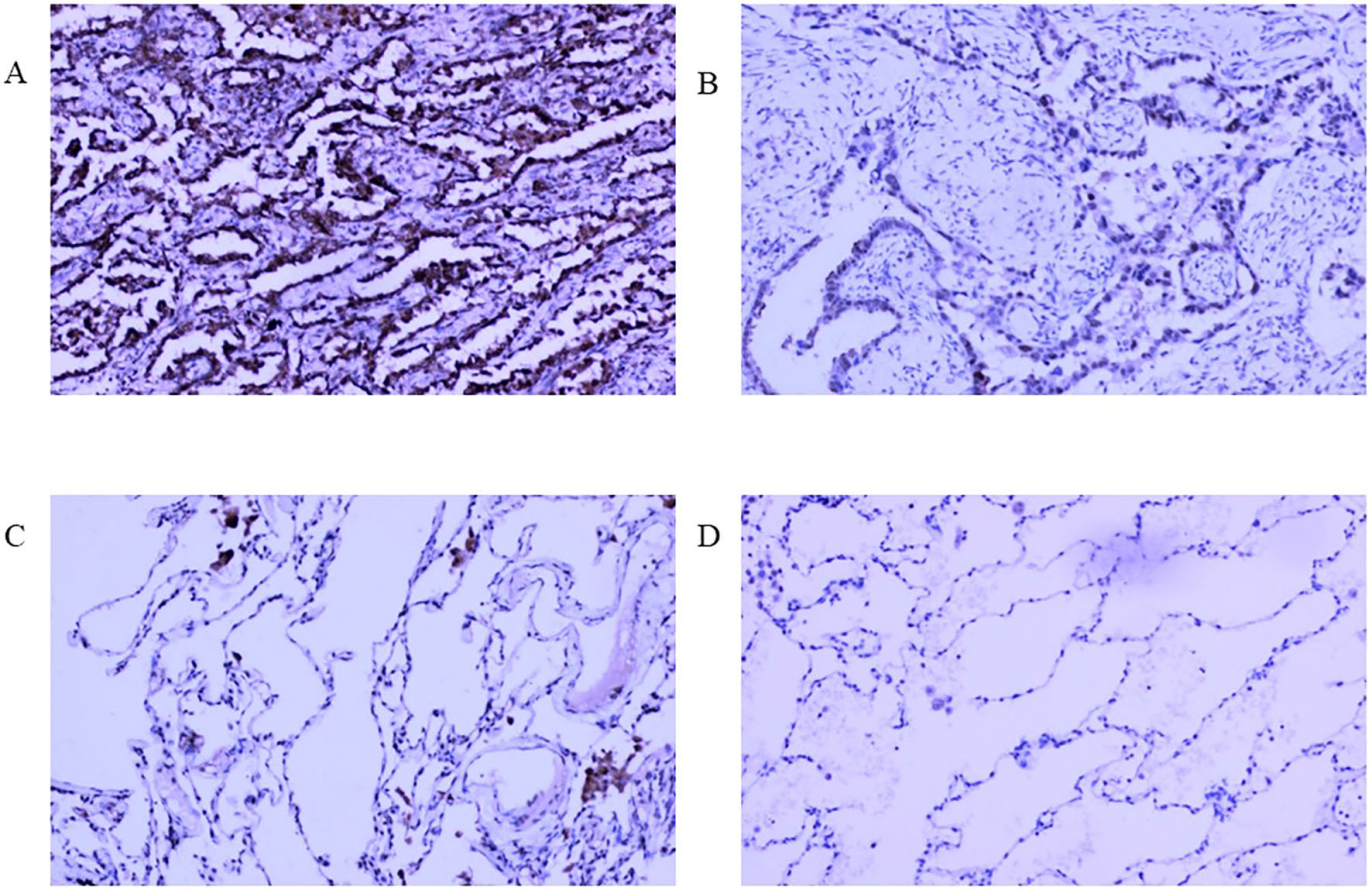
Interpretation of RFC4 in immunohistochemical staining from real-world cohort. **(A)** High expression of RFC4 in LUAD cancer tissue. **(B)** Low expression of RFC4 in LUAD cancer tissue. **(C)** Positive expression of RFC4 in normal lung tissue. **(D)** Negative expression of RFC4 in normal lung tissue.

In our real-world cohort, among 31 cases of LUAD tissue, 24 cases had a strong positive RFC4 expression, 7 cases had a weak positive expression, and no cases demonstrated a negative expression. In adjacent tissues, only six cases showed strong positive RFC4 expression, 10 cases showed a weak positive expression, and 15 cases showed a negative expression. Thus, the strong positive expression rate of RFC4 in LUAD tissues was 77.42% (24/31), whereas that in adjacent tissues was 19.35% (6/31), and the difference was statistically significant (χ^2^ = 26.329, *P*<0.001). An IHC schematic of RFC4 protein in LUAD and normal lung tissues from our real-world cohort is shown in **Figures 13A–D**.

## 4 Discussion

This study has made several important discoveries. First, both RFC4 mRNA and protein are overexpressed in LUAD cancer tissues, indicating a strong correlation between RFC4 and occurrence of LUAD cancer. Second, high RFC4 expression levels were associated with poor prognosis in patients with LUAD. Third, a relationship between the expression status of RFC4 and TIME remodeling was identified. Finally, patients with LUAD with high RFC4 expression levels may be more likely to benefit from immunotherapy.

In the past two decades, cancer research has significantly progressed, with targeted and immunotherapy drugs being constantly updated. The emergence of ICIs, used alone or in combination with chemotherapy, marks a milestone in the treatment of advanced LUAD (28). With the application of an increasing number of ICIs against PD-1, PD-L1, and CTLA-4, more treatment options are available and prognoses in patients with advanced LUAD has significantly improved (29). However, the current effective of immunotherapy for LUAD is still <40% (30). The response of LUAD to these therapies varies greatly, from patients with complete and long-term remission of metastatic diseases to those who rapidly progress and die from cancer despite the use of the latest ICIs. Thus, if patients with LUAD are not effectively selected, many will receive unnecessary and ineffective immunotherapy (31, 32). Unfortunately, biomarkers for predicting the effectiveness of immunotherapy in human cancer are currently lacking. Therefore, new robust markers still need to be explored to guide clinical treatment decisions about ICIs.

RFCs are composed of the following five subunits: RFC1, RFC2, RFC3, RFC4, and RFC5 (16). RFCs not only increase the affinity between DNA polymerase and primer ends, but also reduce the number of PCNAs required to activate DNA polymerase (33). The RFCs exhibit DNA-dependent ATPase activity, which is necessary to activate DNA polymerase (33). The RFC complex contains a new 5′ DNA binding site responsible for transferring the 9-1–1 heterotrimeric clamp onto DNA, playing a role in DNA break repair (34). The role of RFCs in cancer progression has attracted increasing attention (10, 35). RFCs exhibit biological activities in various malignant tumors and may play important roles in the proliferation, progression, invasion, and metastasis of cancer cells (36, 37). Until recently, the role of RFC4 in cancer progression remained underexplored or unclear. Many studies have shown that RFC4 can promote tumor progression and metastasis in lung, nasopharyngeal, hepatocellular and colorectal cancers (16, 17, 36, 37). RFC4 is a regulatory protein that is primarily present in the nucleus (38). RFC4 exists mainly in the RFC complex of DNA and participates in the formation of DNA replication complexes to initiate the replication process of DNA. RFC4 is also involved in various important cellular processes, including DNA strand extension, DNA repair, and the other important signaling pathways (39). To elucidate the mechanism of RFC4 in LUAD, we used a series of bioinformatics methods to comprehensively analyze the gene expression and clinical characteristics of RFC4 in LUAD as well as the relationship between RFC4 expression and survival, microsatellite instability, and immune infiltration.

To the best of our knowledge, this study is the first to investigate the expression levels of RFC4 in cancer and normal tissues using the TCGA, GEO, and HPA databases. RFC4 expression was significantly upregulated in LUAD, which is consistent with its expression in other cancers (10, 16, 36). Thus, RFC4 may be involved in LUAD development and may be an important genetic diagnostic marker for LUAD. Interestingly, the increase in RFC4 expression was highly correlated with the mortality status of patients with LUAD, with RFC4 expression significantly elevated in deceased patients. Further survival analysis suggested that a high RFC4 expression level was an important prognostic factor. Unfortunately, we did not observe any correlation between RFC4 expression levels and tumor staging, nor were they associated with clinical features such as gender, age, and smoking status. Based on these results, RFC4 may be a potential prognostic biomarker of LUAD, providing a new targeted therapy strategy for the treatment of LUAD.

Changes in TIME are important features of tumors, which are highly correlated not only with cancer prognosis but also with tumor response to immunotherapy (40, 41). Several types of immunotherapies, including adoptive cell transfer and ICIs, have achieved long-lasting clinical responses, with the core mechanism of reshaping the TIME, enhancing tumor response to immunotherapy, and promoting tumor cell apoptosis (41). However, the high heterogeneity and dynamism of TIME hinder the precise isolation of immune cells within tumors, making it difficult to comprehensively analyze cancer prognosis. To further investigate the potential value of RFC4 in LUAD, we explored the correlation between RFC4 expression, immune cell infiltration and immunomodulators.

Among the selected immunostimulators, RFC4 expression was positively associated with 14 immunostimulatory factors and negatively correlated with 13 immunostimulatory factors. These results hindered the understanding of the role of RFC4 as an immunostimulator. Therefore, to clarify and summarize the results, we used GSVA to predict the gene set of immunostimulators. The GSVA results showed that RFC4 was negatively correlated with GSVA.Meta (rho=-0.164, *P*<0.001). The same method was applied to immune inhibitors. The GSVA result showed that RFC4 was negatively correlated with GSVA.Meta with no statistical significance (rho=-0.068, *P*=0.122). Based on these results, we inferred that RFC4 mainly alters the TIME by suppressing the expression of immune stimulatory factors. The dual positive/negative correlations between RFC4 and immunostimulators (as shown in **Figure 7A**) suggest that the mechanism by which RFC4 regulates the TIME is very complex. TIME is a complex system with highly precise regulatory mechanisms, and complex crosstalk with stromal components may be key to maintaining the orderly operation of these complex components (42, 43). A focused study on how RFC4 simultaneously suppress and activate immune pathways is required.

In addition, although the bioinformatics results were robust, the biological implications of RFC4 overexpression in LUAD remain unexplored. The present study is an exploratory study; therefore, we cannot yet determine the mechanism by which RFC4 affects the TME, which requires further research. In our preliminary results, RFC4 expression was positively correlated with PD-L1(CD274, as shown in **Figure 7B**), but not with CTLA-4 expression. We speculated that RFC4 might be related to PD1/PD-L1 pathway. Although the GO/KEGG analyses highlight the association of RFC4 with DNA repair and cell cycle pathways, direct mechanistic links to immune evasion (e.g., via PD-L1 regulation or antigen presentation) remain speculative. Therefore, our future study will verify the effect of RFC4 on PD-L1 expression through overexpression or knockdown, demonstrate the direction of gene regulation, and clarify whether RFC4 is a driver or consequence of immune evasion. The RFC4/NOTCH1 signal feedback loop was identified and revealed the mechanism of RFC4 promoting NSCLC metastasis and stemness, indicating its therapeutic and diagnostic/prognostic potential for NSCLC treatment (17).

With breakthroughs in tumor-related immunosuppressants and immunostimulants, ICIs have been widely used in tumor immunotherapy and have achieved significant and encouraging results. ICIs targeting PD-1/PD-L1 have been approved for the treatment of various malignant tumors, including melanoma, lymphoma, LC, and many other cancers. Therefore, speculating that RFC4 expression may regulate the infiltration level of tumor immune cells and the immune response, ultimately affecting the prognosis of patients with cancer, is reasonable. To verify this hypothesis, we employed two methods to explore the impact of RFC4 expression on immune cell infiltration into the TME. Using the CIBERSORT algorithm, we found that T cells CD4 memory resting, mast cells resting, dendritic cells resting, monocytes, macrophages M2, and plasma cells were negatively correlated with RFC4 gene with significance, whereas T cells gamma delta, mast cells activated, T cells CD4 memory activated, macrophages M0, T cells follicular helper, T cells CD8, macrophages M1 were positively correlated with RFC4 gene. Using the ESTIMATE algorithm, we observed that cancer tissues in the RFC4^High^ group had lower stromal and immune scores, lower ESTIMATE scores, and higher tumor purity. Based on the above analysis results, we confirmed that RFC4 played an important role in reshaping TIME. Therefore, the targeted regulation of RFC4 expression may alter the TIME of patients with LUAD and achieve better immunotherapy outcomes. Immunotherapy predictions further confirmed our hypothesis. In the TIDE algorithm, lower TIDE scores were observed in the RFC4^High^ group, indicating higher immune sensitivity and increased patient benefit from ICI treatment. Moreover, high RFC4 expression levels were correlated with lower dysfunction and higher exclusion. Furthermore, high RFC4 expression levels were correlated with higher MDSC, CAF, and CD8 scores and lower TAM.M2 and IFNG scores. In tumors with a high infiltration of immune cells, dysfunctional effector toxic T cells can effectively kill tumor cells; however, their function is suppressed for some situation. Lower dysfunction and higher exclusion indicated that the cancer environment is more suitable for immunotherapy.

Our study also predicted the impact of RFC4 on 14 biological functions in common cancers. RFC4 is widely involved in other biological processes, including angiogenesis, apoptosis, cell cycle, differentiation, DNA damage, DNA repair, hypoxia, inflammation, invasion, proliferation, quiescence and stemness. According to previous reports, RFC4 is mainly related to DNA replication and repair, and our research yielded similar results, indicating that our prediction results have highly accurate (11, 37, 39). The manner by which RFC4-driven DNA replication/repair processes intersect with immune evasion requires further investigation. In most cancer tissues, the expression of DNA replication/repair genes is high (44), which may reflect the proliferative properties of cancers. High expression of DNA replication/repair genes is commonly a passive physiological state, although it is crucial for maintaining genomic stability. Genomic instability is a hallmark of cancer cell differentiation from normal cells (45). Genomic instability is an important genetic feature of changes in the TIME (46). In the presence of ATP, RFC4 can assemble PCNA and DNA polymerase δ onto a template using primers, thereby effectively extending the DNA replication strand. This process is essential for DNA replication and repair. Therefore, RFC4 expression was highly correlated with DNA repair.

Our findings strengthen the idea that high RFC4 expression levels were associated with poor prognosis in patients with LUAD. From the perspective of bioinformatics analysis, RCF4 alters tumor prognosis by reshaping the TIME, and targeted inhibition of RCF4 may be a promising new strategy for the treatment of LUAD. Our study has some limitations. First, our study was based only on the TCGA and GEO databases and should be validated in clinical cohorts in the future. Second, our study relied on TIDE scores to predict immunotherapy response but lacked experimental validation. In the future, a real-world cohort should be collected to verify whether RFC4 expression correlates with immunotherapy responses. Third, our study identified RFC4 as a potential therapeutic target but did not provide functional validation. In the future, silencing (shRNA/CRISPR) or overexpression of RFC4 in LUAD cell lines could be used to examine its impact on tumor growth, immune evasion, drug sensitivity, and cytokine expression. Fourth, the biological function of RFC4 protein expression in LUAD cells should be experimentally validated. Further exploration of the factors and upstream and downstream signaling pathways that regulate RFC4 *in vivo* is required. Finally, the biomarkers currently established, such as PD-L1 expression and tumor mutation burden, have shown very optimistic predictive value in immunotherapy (47, 48). It is still unclear whether RFC4 can provide additional predictive value compared with PD-L1 expression and tumor mutation burden. Further exploration is required in real-world cohorts.

## 5 Conclusion

RFC4 may be a promising biomarker for tumorigenesis and could effectively predict immunotherapy response in LUAD. RCF4 altered tumor prognosis by reshaping the TIME, and targeted inhibition of RCF4 may be a promising new strategy for the treatment of LUAD.

## Data Availability

The original contributions presented in the study are included in the article/**Supplementary Material**. Further inquiries can be directed to the corresponding author.

## Supplementary material

Supplementary Figure 1 Analysis of tumor-infiltrating immune cells changes in different RFC4 status in LUAD cohort via CIBERSORT. **(A)** Cell proportion of 22 immune cells between RFC4High group and RFC4Low group. **(B)** Cell proportion of 22 immune cells in all LUAD cancer samples.
