# Supplementary figures and images for: Comprehensive analysis of RFC4 as a potential biomarker for regulating the immune microenvironment and predicting immune therapy response in lung adenocarcinoma
Source: Front Immunol. 2025 Jun 19;16:1578243. doi: 10.3389/fimmu.2025.1578243 (PMC12222213; doi:10.3389/fimmu.2025.1578243)

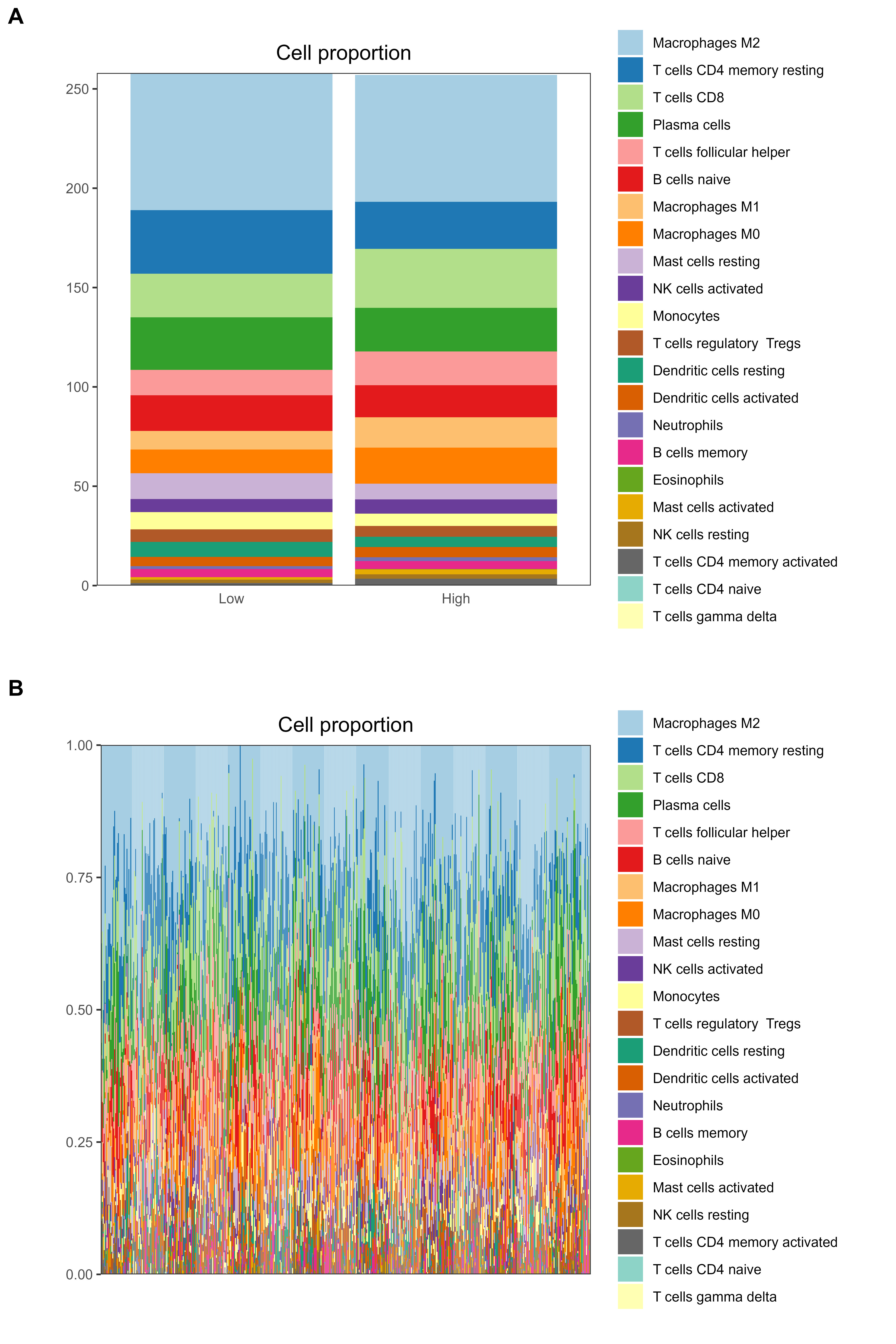

Supplement: Supplementary Figure 1 — Analysis of tumor-infiltrating immune cells changes in different RFC4 status in LUAD cohort via CIBERSORT. (A) Cell proportion of 22 immune cells between RFC4High group and RFC4Low group. (B) Cell proportion of 22 immune cells in all LUAD cancer samples. [file Image1.jpeg]
